# Aramid Nanofibers/Reduced Graphene Oxide Composite Electrodes with High Mechanical Properties

**DOI:** 10.3390/nano13010103

**Published:** 2022-12-25

**Authors:** Jingyi Wang, Shaojie Lu, Mingyu Ye, Xiaowan Zhan, Hongbing Jia, Xin Liao, Antonio Francisco Arcanjo de Araújo Melo

**Affiliations:** 1 School of New Materials and Shoes & Clothing Engineering, Liming Vocational University, Quanzhou 362000, China; 2 Key Laboratory for Soft Chemistry and Functional Materials of Ministry of Education, Nanjing University of Science and Technology, Nanjing 210094, China; 3 São Carlos Institute of Physics, University of São Paulo, São Carlos 13560-970, Brazil; 4 Materials Engineering Graduate Program, Federal Institute of Education, Science and Technology of Piauí, Teresina 64000-040, Brazil

**Keywords:** aramid nanofibers, reduced graphene oxide, flexible film electrodes, mechanical properties

## Abstract

In this work, aramid nanofibers (ANFs)/reduced graphene oxide (ANFs/RGO) film electrodes were prepared by vacuum-assisted filtration, followed by hydroiodic acid reduction. Compared with thermal reduced ANFs/RGO, these as-prepared film electrodes exhibit a combination of mechanical and electrochemical properties with a tensile strength of 184.5 MPa and a volumetric specific capacitance of 134.4 F/cm^3^ at a current density of 0.125 mA/cm^2^, respectively. In addition, the film electrodes also show a superior cycle life with 94.6% capacitance retention after 5000 cycles. This kind of free-standing film electrode may have huge potential for flexible energy-storage devices.

## 1. Introduction

Electric power is an environment-friendly energy source, and supercapacitors are important devices for this clean energy storage [1,2,3,4,5,6]. Due to their high-power density, long durability, and moderate energy density, supercapacitors have been widely considered to manufacture lightweight, flexible, and portable electronic devices [7,8]. Over the past decades, numerous research has focused on the preparation of flexible electrodes, since flexible electrodes are the key to the construction of flexible supercapacitors [9,10,11], which can be in smart wearable devices [12,13,14].

Graphene is a two-dimensional (2D) carbon-based nanosheet which is composed of sp^2^-bonded carbon in a honeycomb crystal lattice. Due to its super properties of excellent electrical conductivity, large surface area and favorable mechanical strength, graphene has been widely used in supercapacitors electrodes [15,16,17]. The macroscopic assembly prepared by graphene, such as graphene film, is an ideal material for free-standing flexible electrodes [18,19]. However, the irreversible restacking of 2D graphene sheets will lead to poor mechanical properties during assembly process, resulting from the lack of stress transfer [20,21]. Therefore, it is necessary for flexible supercapacitors to the prepare graphene free-standing electrode materials with superior mechanical and electrochemical properties.

Aramid nanofibers (ANFs), which are derived from a high-performance Kevlar, have been considered as novel nanoscale building blocks for reinforcement of composite materials [22,23]. Making the most of the excellent mechanical properties of Kevlar (Young’s modulus of 90 GPa and tensile strength of 3.6 GPa), ANFs are extensively applied in the fabrication of macro-films and hydrogels with excellent mechanical properties [24,25,26,27]. It has been demonstrated that the present of ANFs in reduced graphene oxide (RGO) film electrode can achieve excellent balance between mechanical properties and capacitance performance. For example, Kwon et al. [28] prepared ANFs/RGO composite film via vacuum-assisted filtration, followed by thermal reduction in vacuum. Benefiting from the synergistic effect, the resulted ANFs/RGO film electrode can reach a moderate tensile strength of 100.6 MPa and volumetric specific capacitance of 83 F/cm^3^. However, due to the vigorous release of gas (i.e., CO_2_) caused by thermal decomposition during the reduction process, the thermal reduction of GO composite film fails to yield a uniform structure, leading to defects and holes inside the film [29]. It is worth pointing out that the defects and holes inevitably serve as stress concentration points and induce the rapid growth of cracks along the whole film, resulting in the relatively limited absorbed fracture energy and mechanical properties. In contrast, the utilization of hydroiodic (HI) acid for reducing GO is more suitable to obtain RGO composite film with high mechanical properties, since it can produce more compact and intact layered architecture based on the nucleophilic substitution reaction [30]. Therefore, it is prospective to prepare ANFs/RGO film electrodes with higher mechanical properties by reduction with HI without damaging the electrochemical performance.

In this work, we have fabricated ANFs/RGO film electrodes through vacuum-assisted filtration, followed by reduction with HI. The obtained film electrodes accomplish the combination of high mechanical properties and electrochemical performance. The tensile strength of ANF/RGO film electrodes reach as high as 184.54 MPa, higher than the reported ANF/RGO film electrode by thermal reduction (tensile strength of 100.6 MPa) [28]. Furthermore, ANF/RGO film electrodes obtain a volumetric specific capacitance of 134.4 F/cm^3^ at a current density of 0.125 mA/cm^2^ and a cycle life with 94.6% capacitance retention after 5000 cycles. This work brings significant contributions to the construction of flexible supercapacitors.

## 2. Experimental Method

Kevlar 49 yarn was purchased from DuPont Company. Graphite powder (average size ≤ 30 μm) was purchased from Nanjing Chemical Reagent Co. Ltd., Nanjing, China. Phosphoric acid (H_3_PO_4_), hydroiodic (HI) acid, polyvinyl alcohol (PVA, *M*_w_ 145000) and potassium permanganate (KMnO_4_) were purchased from Aladdin Reagent Co. Ltd., Shanghai, China. Sulfuric acid (H_2_SO_4_), nitric acid (HNO_3_), hydrochloric acid (HCl), hydrogen peroxide (H_2_O_2_, 30%w/w), potassium hydroxide (KOH), and dimethyl sulfoxide (DMSO) were purchased from Sinopharm Chemical Reagent Co. Ltd., Shanghai, China. All reagents were used as received without further purification. Water used throughout all experiments was purified by deionization and filtration through a Millipore system.

The preparation of ANFs dispersion was conducted according to the work of Kotov [25]. In brief, 1.0 g of Kevlar 49 yarn and 1.5 g of KOH were added into 500 mL of DMSO. The mixture was magnetically stirred for 1 week at room temperature, and the dark red ANFs/DMSO dispersion (2 mg/mL) was finally obtained.

Hence, 3 g of graphite powder was put into a mixture of 360 mL of H_2_SO_4_ and 40 mL H_3_PO_4_. Then, 18 g of KMnO_4_ was slowly added to the mixture, followed by stirring at 50 °C for 12 h. Then, 400 mL of ice and 3 mL of 30 wt% H_2_O_2_ were poured into the mixture. The mixture was washed with 5 wt% HCl solution and the supernatant was decanted away. The filtered mixture was centrifuged and washed for several times until it was neutral. The graphite oxide powder was obtained by vacuum freeze-drying of the mixture. GO powder was dispersed in deionized (DI) water and exfoliated to obtain 2mg/mL GO dispersion by ultrasonication. 100 mL of DMSO was added to the GO dispersion and further sonicated for 1 h. Then, a GO/DMSO dispersion (1 mg/mL) was finally obtained via solvent exchange.

ANFs/RGO film electrodes were fabricated via vacuum-assisted filtration, followed by reduction with HI. First, ANFs/DMSO dispersion (2 mg/mL) was diluted to 1 mg/mL. Then, the desired amount of GO/DMSO dispersion (1 mg/mL) was added into the ANFs/DMSO dispersion and stirred for 1 h. After stirring, the mixture was heated to 80 °C. For the mixture, a certain amount of DI water (1 mL of DI water for every 1 mg of ANFs) was added and further stirred for 2 h to re-protonate ANFs, which resulted in the formation of a gel-like system. Then, the mixture was vacuum filtered on a nylon membrane (0.22 μm pore size). The obtained ANFs/GO films were then washed with DI water three times and dried in air. Then, the ANFs/GO films were peeled off from the filter membrane and dried at 80 °C in a vacuum for 72 h. Finally, ANFs/GO films were immersed into HI solution for 2 h to yield ANFs/RGO films, and then washed repeatedly with ethanol to remove the residual HI. The total solid mass in the mixture was kept at 24 mg. Thus, a series of ANFs/GO films with different ANFs loadings can be obtained and named as nA-GO (n represents mass percent of ANFs content). Similarly, ANFs/RGO films were expressed as nA-RGO.

Flexible solid-state supercapacitor devices based on ANFs/RGO films were fabricated in symmetrical two-electrode configuration [31]. Briefly, 3 g PVA and 3 g H_2_SO_4_ was dissolved in 30 mL DI water, then vigorous stirred at 85 °C to obtain transparent solution. After the solution cooled down, PVA/H_2_SO_4_ gel electrolyte was obtained. Next, two pieces of the nA-RGO films were immersed in the PVA/H_2_SO_4_ gel electrolyte for 5 min and then assembled into a supercapacitor. In this process, PVA/H_2_SO_4_ gel membrane acted as an electrolyte and a separator. The devices were then placed in a fume hood at room temperature to evaporate the excess water. The average thickness values of electrodes are 14 μm, 1.0 μm, 0.9 μm, and 0.8 μm for RGO, 10A-RGO, 25ARGO, and 50A-RGO film electrodes, respectively. The typical combined mass of a pair of electrodes was around 10 mg. The scheme of solid-state supercapacitor devices based on ANFs/RGO films is showed in Figure S1.

The morphologies of the samples were characterized on scanning electron microscopy (SEM) (JSM-6380LV, JEOL Co. Ltd., Tokyo, Japan). Raman spectra were recorded with a Raman spectrometer (inVia-H31894, Renishaw Corporation, Wotton-under-Edge, UK) at an excitation wavelength of 514 nm. X-ray diffraction (XRD) patterns were carried on a D8-Advanced X-ray diffractometer (Bruker, Bremen, Germany) with Cu Kα radiation (λ = 0.154 nm) from 5° to 50°, at a scanning speed of 5 °/min. X-ray photoelectron spectroscopy (XPS, PHI Quantera II, Tokyo, Japan) was performed to further study the chemical bonds and elemental compositions. The electrical conductivity of film electrodes was measured using a Keithley 2400 multiple-function source-meter (TEKTRONIX, INC., Beaverton, OR, USA) with a four-probe method at room temperature. The stress–strain testing of film was performed using a CMT-4254 universal tensile testing machine (MTS Co., Ltd., Wan Chai, Hong Kong) at room temperature with a humidity of 25%. The samples were cut into strips of 30 × 3 mm. The tensile rate was 0.5 mm/min with a gauge distance of 20 mm. The test results were averaged over five independent measurements. The surface wettability of the films was determined by a contact angle tester (SL200B, Solon Tech (Shanghai) Co. Ltd., Shanghai, China).

The electrochemical measurements were carried out on an Autolab-PGSTAT 302F workstation (Metrohm, Switzerland). Cyclic voltammetry (CV) and galvanostatic charge-discharge (GCD) measurements were conducted from 0 V to 0.8 V vs. Ag/AgCl in 1 M H_2_SO_4_.

For three-electrode tests, the volumetric specific capacitance (*C*_v_) was calculated using the follow equation:

(1) $$C_{v}=\frac{I\Delta t}{v\Delta V}$$

where *I* is the discharge current (A), Δ*t* is the discharge time (s), *v* is the volume of film electrode (cm^3^), and Δ*V* is the potential window (V).

The *E* is volumetric energy (mWh/cm^3^) and *P* is power density (mW/cm^3^) of supercapacitors, which can be calculated as following:

(2) $$E=\frac{C_{v}\times \Delta V^{2}}{2\times 3600}$$

(3) $$P=3600\times \frac{E}{t}$$

where *C*_v_ is the volumetric capacitance of the device (F/cm^3^), Δ*V* is the potential window (V), *t* is the discharge time (s).

## 3. Results and Discussion

**Figure 1:**
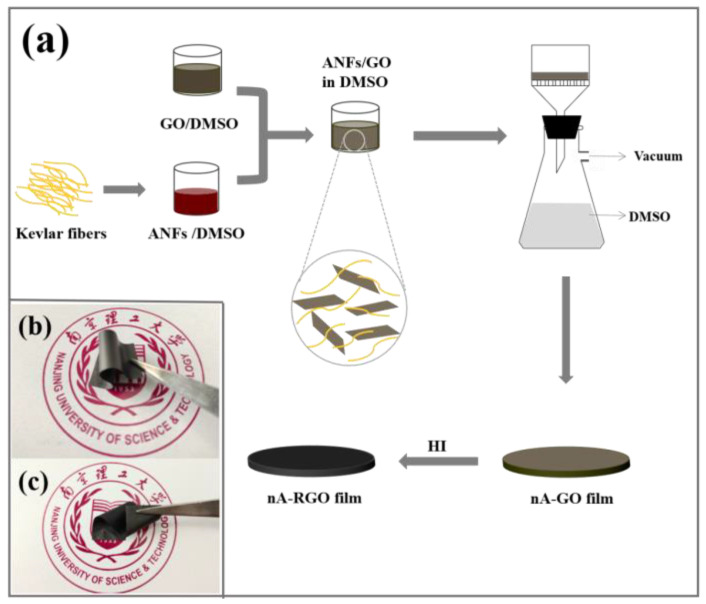
(**a**) Schematic of the fabrication process for nA-RGO film electrode. (**b**,**c**) Digital images of nA-RGO free-standing film electrode for bending and folding.

nA-RGO film electrodes were fabricated via vacuum-assisted filtration, followed by reduction with HI, as illustrated in Figure 1a. GO/DMSO dispersion was prepared via solvent exchange, where the GO was negatively charged [32]. The ANFs /DMSO dispersion was formed by dissolving macro-sized Kevlar 49 yarns in DMSO. In this stage, the amide groups of the PPTA backbone suffered a deprotonation process, forming a stable and dark red dispersion containing negatively charged nanofibers [25]. Subsequently, the ANFs/DMSO and GO/DMSO dispersion were mixed and stirred. A certain amount of DI water was slowly added into the mixture of GO/ANFs/DMSO to induce the re-protonation process of ANFs, which allowed hydrogen bonding between ANFs and GO sheets [33]. The nA-GO composite film was obtained by removing solvent from the mixture using vacuum filtration. Finally, HI was used to chemically reduce the composite film into conductive nA-RGO film electrode. In this process, the composite film transformed from dark brown to black in color. It can be observed from Figure 1b,c that the resulted nA-RGO film electrode could withstand different degrees of deformation, such as bending and folding without breaking, indicating a promising prospect of nA-RGO film electrode for the fabrication of flexible supercapacitors.

**Figure 2:**
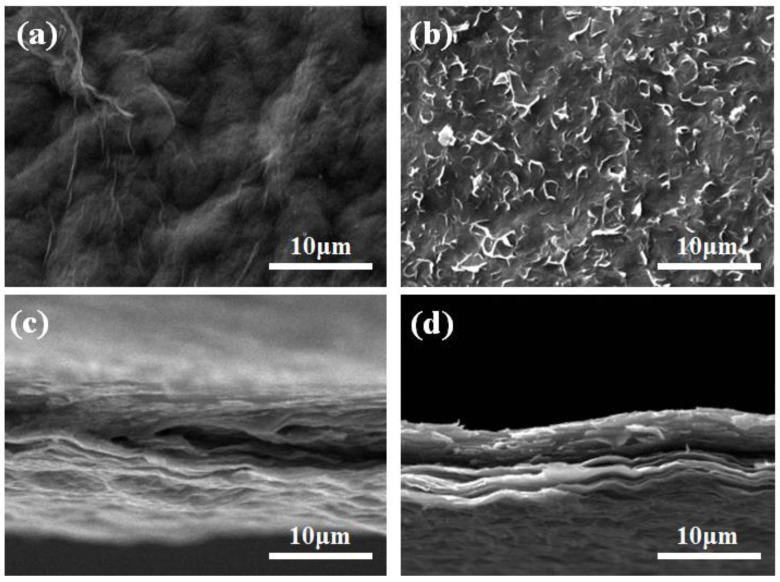
Surface SEM photograph of (**a**) RGO film, (**b**) 25A-RGO film. Cross-sectional SEM photograph of (**c**) RGO film, (**d**) 25A-RGO film.

Figure 2a,b exhibit the surface morphology SEM images of RGO and 25A-RGO films, respectively. The surface of RGO film shows typical wrinkled morphology, while the surface of 25A-RGO film (Figure 2b) is flatter and smoother than that of RGO film [34]. This indicates that stronger π–π interaction between the RGO sheets and ANFs improves the structural integrity of the 25A-RGO film. Cross-sectional SEM images of RGO and 25A-RGO films (Figure 2c,d) show that both films possess typical layered structure. Additionally, compared with the compact packing of RGO sheets in the RGO film (Figure 2c), the interlayer gap of the 25A-RGO film is larger (Figure 2d), which can be attributed to the intercalation of ANFs, contributing to the accessibility of electrolyte ions [35,36].

**Figure 3:**
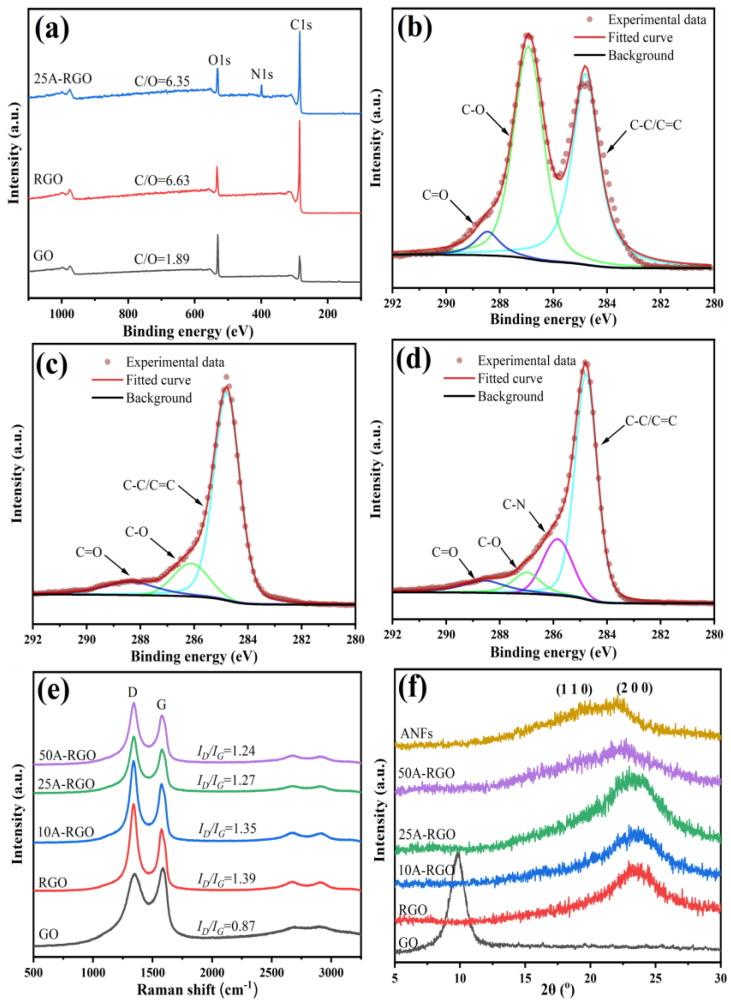
XPS spectra of GO, RGO and 25A-RGO films (**a**) survey scan spectrum and (**b**–**d**) high-resolution C 1s spectra. (**e**) Raman spectra of GO, RGO and nA-RGO films. (**f**) XRD patterns of GO, ANFs, RGO and nA-RGO films.

Figure 3a shows the XPS survey spectra of GO, RGO and 25A-RGO films, and the C/O atomic ratio increases from 1.89 to 6.63 after chemical reduction of GO by HI, suggesting the removal of a large fraction of oxygenated groups. Regarding the high-resolution XPS C 1s spectra (Figure 3b,c), the observed characteristic peaks are ascribed to C-C/C=C (284.8 eV), C-O (286.9 eV), and C=O (288.3 eV), respectively. Remarkably, the signal arising from the C-O moieties decreases greatly, which further demonstrates the efficient elimination of the oxygen-containing groups on the GO basal plane, accompanying with the partially restored conjugated structures [37,38]. For the 25A-RGO composite film, observation of a new N 1s peak in the survey spectrum of 25A-RGO film provides direct evidence for the presence of ANFs, which is also reflected by the C-N group (285.7 eV) that appeared in the high-resolution C 1s spectrum (Figure 3d).

Raman spectra of GO, RGO, and nA-RGO films are illustrated in Figure 3e. In the Raman spectrum of GO film, there are two characteristic peaks at 1344 cm^−1^ and 1591 cm^−1^, corresponding to the D-band caused by the defects and the G-band caused by the first-order scattering of the E_2g_ mode, respectively [39]. The intensity ratio of the D-band to the G-band (*I_D_/I_G_*) of RGO film increases to 1.39 compared to that of GO (0.87), which demonstrates the restoration of sp^2^ carbon and decrease in the average sizes of the sp^2^ domains upon reduction of GO [40,41]. Additionally, the *I_D_/I_G_* decreases from 1.39 to 1.24 with the increase of ANFs content in nA-RGO films, reflecting the strong π–π interaction between ANFs and RGO sheets [42].

The crystallographic structure of the composite films is characterized by XRD patterns, as shown in Figure 3f. A sharp diffraction peak at 2*θ* = 9.82° is detected for GO film, corresponding to an interlayer distance of 0.901 nm. After chemical reduction of GO film, a weak and broad peak locates at 23.26° for RGO film, and the interlayer distance is calculated to be 0.382 nm. Such a decrease in the interlayer distance further proves the efficient elimination of the oxygenated groups by HI [43]. The XRD pattern of ANFs shows two diffraction peaks at 2*θ* = 19.66° and 22.08°, corresponding to (110) and (200) crystal planes, respectively [40,41]. For the nA-RGO composite films, the diffraction peak of RGO becomes broader and gradually shifts to smaller angles, which is like the previous report [44]. The corresponding interlayer distances of nA-RGO with smaller diffraction angle increase from 0.382 to 0.397 nm with increasing ANFs content from 0 to 50 wt%. The enlarged interlayer distance within nA-RGO composite film will contribute to increase the electrochemical active sites accessible by the electrolyte ions [45].

**Figure 4:**
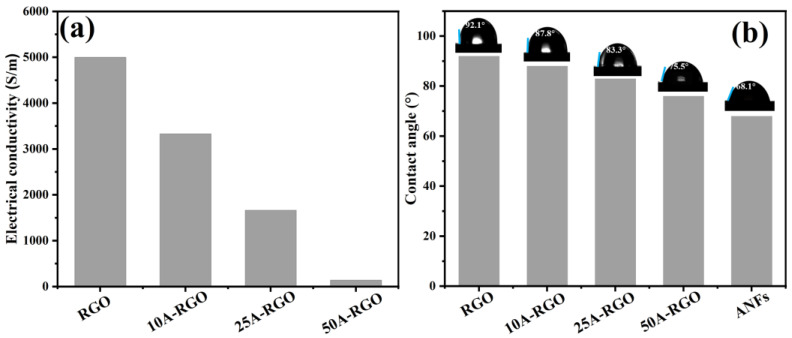
(**a**) Electrical conductivity of RGO and nA-RGO films. (**b**) water contact angles of RGO, ANFs and nA-RGO films.

Figure 4a displays the electrical conductivity of RGO and nA-RGO films. Owing to the electrical insulating nature of ANFs, the electrical conductivity of the nA-RGO composite films gradually decreases with the increase of ANFs content. The electrical conductivity decreases from 5000 S/m for RGO film to 1667 S/m for 25A-RGO. Further increasing the ANFs content to 50%, the electrical conductivity drastically declines to 143 S/m, since the excess ANFs will form many electrically insulating domains in the composite film [42].

Figure 4b shows the static water contact angle values of the film samples, where the wettability of the composite film is improved with the increase of ANFs content. For example, RGO film displays an average contact angle value of 92.1°, while 50A-RGO film presents a much lower value of 75.5°. This decrease in water contact angle can be attributed to the polar functional groups (i.e., amide groups) in the backbone of ANFs, which enhance the hydrophilicity of composite films. The improved wettability of electrode materials is much desired for providing higher electrode utilization efficiency [43].

**Figure 5:**
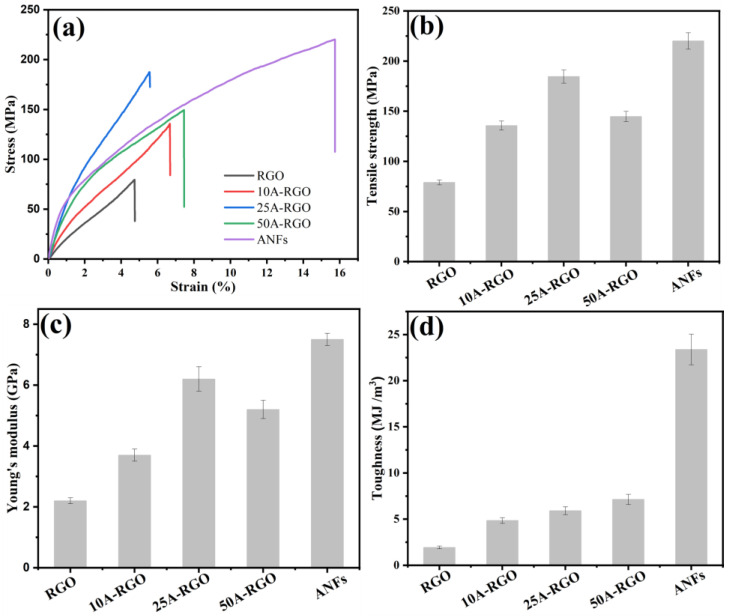
(**a**) Stress-strain curves, (**b**) tensile strength, (**c**) Young’s modulus and (**d**) toughness of Films samples.

Superior mechanical performance is a vital indicator of the flexible electrodes operated in practical applications, especially for the carbonaceous electrodes with intrinsic brittleness. Figure 5a illustrates the stress–strain curves of the film samples, where the mechanical properties of nA-RGO composite films are much superior to that of RGO film. The trends of tensile strength, Young’s modulus, and toughness of film samples with different ANFs loadings are shown in Figure 5b–d. The pure ANFs film has high mechanical properties with tensile strength of 220.0 ± 8.2 MPa and Young’s modulus of 7.5 ± 0.2 GPa. Owing to the weak interactions between RGO sheets, the pure RGO film shows a tensile strength of 79.0 ± 2.4 MPa and a Young’s modulus of 2.2 ± 0.1 GPa, which is consistent with the previous report [29,46,47]. When the ANFs loadings is 25 wt%, the tensile strength and Young’s modulus reach their peaks of 184.5 ± 6.0 MPa and 6.2 ± 0.4 GPa, respectively, which is 2.3 times and 2.8 time higher than those of pure RGO. This remarkably improved mechanical performance may be attributed to the strong interfacial interactions between RGO sheets and ANFs [17] (i.e., hydrogen bonding and π-π interaction, which has also been proved in SEM section), which is favorable to the effective load transfer during stretching [48]. When ANFs loadings is 50 wt%, both the strength and modulus decrease for the composite film, since the poor dispersion of the ANFs in ANFs/RGO composite film, the concentration of stress in vicinity of the ANFs agglomerate may act as a seed point to initiative the formation of cracks resulting in poor mechanical strength. Additionally, the contribution of ANFs to the mechanical properties is further reflected by the steady increment in toughness of composite film with ANFs content (Figure 5d). Notably, here the tensile strength of 25A-RGO film is 83.4% higher than that of reported ANFs/RGO composites with thermal reduction [28]. These differences in mechanical properties can be attributed to the effect of the reduction method on the microstructure of the resultant composite films. During the thermal reduction process, the thermal decomposition of oxygenated groups on GO sheets leads to the vigorous gas release, which induces many defects and holes inside the film [29]. These defects and holes inevitably serve as stress concentration points and induce the quick growth of cracks along the whole film, resulting in the relatively limited absorbed fracture energy and mechanical properties. In contrast, benefiting from the nucleophilic substitution reaction mechanism, 25A-RGO film reduced with HI can yield a uniform and intact architecture [30]. The advantage of reduction with HI is directly reflected by the higher tensile strength of pure RGO film (79.0 ± 2.4 MPa), while the tensile strength of RGO film with thermal reduction is only 34.4 ± 0.1 MPa [28].

**Figure 6:**
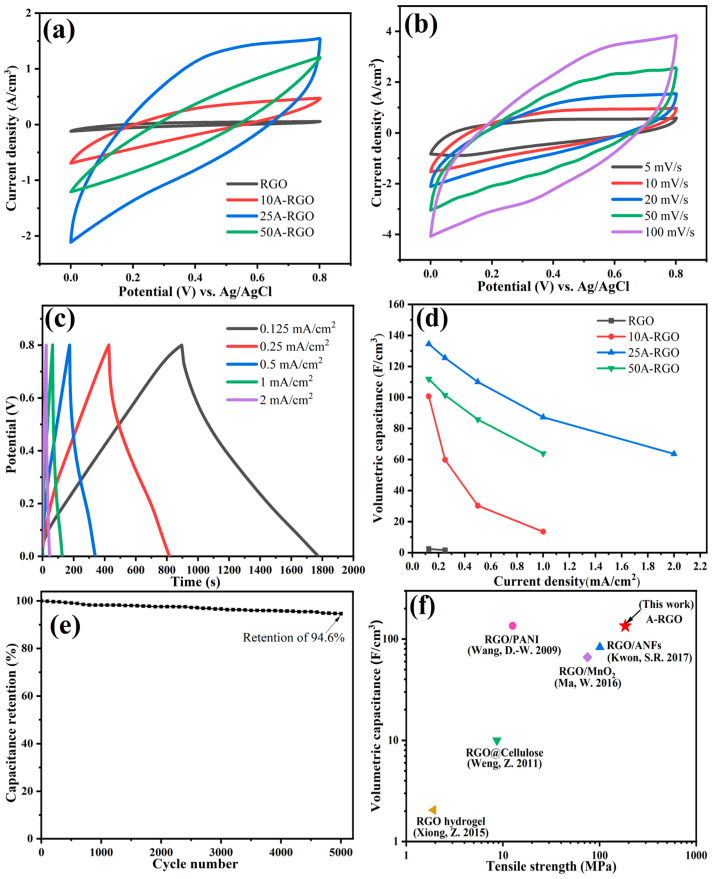
(**a**) CV curves of RGO and nA-RGO film electrodes at 20 mV/s, (**b**) CV curves of 25A-RGO film electrode at different current densities. (**c**) GCD curves of 25A-RGO film electrode at different scan rates, (**d**) volumetric specific capacitances at different current densities of all film electrode and (**e**) cycle behavior of 25A-RGO film at a current density of 2 mA/cm^2^. (**f**) comparison of the volumetric specific capacitance and tensile strength of 25A-RGO film electrode with others [28,52,53,54,55].

Electrochemical properties of the film electrodes are investigated by cyclic voltammetry (CV) and galvanostatic charge-discharge (GCD) measurements with 1.0 M H_2_SO_4_ aqueous solution using a three-electrode system. Figure 6a exhibits the CV curves of RGO and nA-RGO film electrodes at a scan rate of 20 mV/s. The curves of RGO, 10A-RGO and 25A-RGO film electrodes show nearly rectangular shapes, demonstrating a typical electrical double-layer behavior [49]. As the content of ANFs reaches 50 wt%, the curve deviates from rectangular shape, due to the increased internal resistance caused by the excess insulating ANFs [50]. The CV integrated area of nA-RGO film electrodes is much larger than that of pure RGO film, indicating enhanced volumetric specific capacitance (*C*_v_). This can be ascribed to that the intercalated ANFs can potentially act as scaffold to enlarge the interlayer distances between RGO sheets (proved by XRD section) and mitigate RGO sheet aggregation during reduction by HI, which improves the accessibility of electrolyte ions and provides higher electrode utilization efficiency. As proved by adsorption-desorption isotherm curves of RGO and nA-RGO in Figure S2. Compared with RGO, the 25A-RGO has greater BET area, because the ANFs enlarges the interlayer distances between RGO sheets and reduce the assembly of RGO sheets. As a result, the value of BET area increase, and thus the aggregation of RGO decreases, causing the larger integrated area of CV curves. However, the BET value of composite film with 50% ANFs decreases, which may due to the poor dispersion and aggregation of ANFs in composite film, causing less propping up effect on RGO layers. Consequently, the integrated area of CV curves reduces. Figure 6b shows the response of 25A-RGO film electrode as the scan rate varied from 5 mV/s to 100 mV/s. As shown in Figure 6b, the current densities increase with increasing scan rates from 5 to 100 mV/s, and the CV curves keep the same rectangular shape even at a high scan rate of 100 mV/s, suggesting an outstanding capacitance behavior [51]. Figure S3 and Figure 6c show the GCD curves of RGO and composite film electrodes at different current densities. It can be seen that all of the GCD curves exhibit nearly triangular shape, demonstrating the typical electrical double-layer capacitive characteristic. For RGO film, the current density can reach only 0.25 mA/cm^2^, due to the dense structure of RGO film causing by the self-stacking of reduced graphene. This dense structure of film can be enlarged by adding ANFs, and enables electrolyte ions to easily penetrate into the inner of electroactive materials during charge-discharge [31]. All the volumetric specific capacitances (*C*_v_) of film electrodes at different current densities are shown in Figure 6d. In addition, the areal capacitance (*C*_a_) and gravimetric capacitance (*C*_g_) of RGO and nA-RGO film electrodes at different current densities are also shown in Figures S4 and S5. The pure RGO film only exhibits a *C*_v_ of 2.2 F/cm^3^ at a current density of 0.125 mA/cm^2^. In contrast, the introduction of ANFs significantly enhances the *C*_v_ of composite film electrodes since the presence of ANFs enlarges the interlayer distances between RGO sheets and shortens the ion diffusion distance. Consequently, the *C*_v_ value of ANFs/RGO composites is higher than that of pure RGO film. Further increasing the ANFs content, the *C*_v_ declines for 50A-RGO film electrode, resulting from the unfavorable electron transfer caused by the decreased conductivity. Similarly, the *C*_a_ and *C*_g_ of ANFs/RGO composite films are higher than those of the pure RGO film electrode. Long cycle life is an important factor for supercapacitor electrodes during practical applications. Taking 25A-RGO film electrode as an example, the cycle stability of 25A-RGO film electrode at a constant current density of 2 mA/cm^2^ is shown in Figure 6e. The film electrode delivers capacitance retention of 94.6% after 5000 cycles, indicating its superior cyclic stability and high reversibility. The coulombic efficiencies are shown in Figure S6a. A comparison of the volumetric specific capacitance vs. tensile strength between our work and other free-standing film or paper electrodes reported in the literature is displayed in Figure 6f [28,52,53,54,55,56,57,58]. The strength and *C*_v_ of the 25A-RGO film electrode are superior to the electrical double-layer electrode materials and show the comparability with those of pseudo capacitive electrode materials. Clearly, the plot shows that the 25A-RGO composite film in this work exhibits a good combination of electrochemical and mechanical properties.

**Figure 7:**
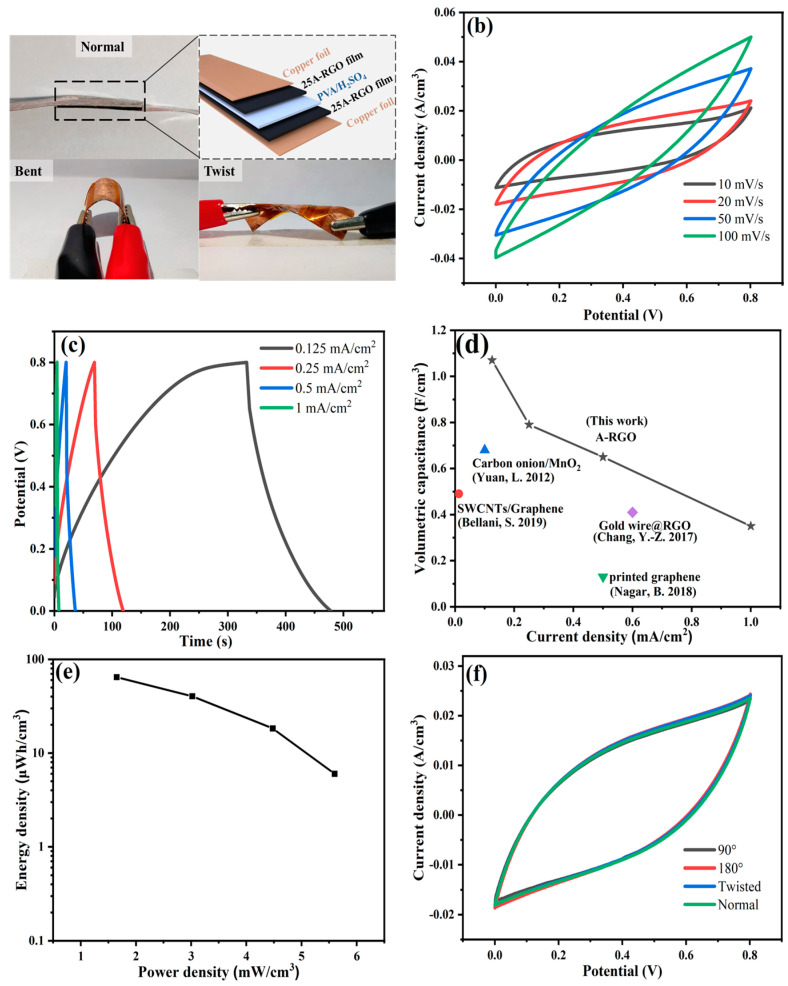
(**a**) Schematic structure and digital images of the 25A-RGO solid-state supercapacitor device, (**b**) CV curves of the device at different scan rates and (**c**) GCD curves of the device at different current densities. (**d**) Comparison of volumetric specific capacitance of 25A-RGO composite film-based supercapacitors with the previously reported supercapacitors. (**e**) Ragone plot of 25A-RGO solid-state supercapacitor device, (**f**) CV curves for the device in bent and twisted at a scan rate of 20 mV/s [60,61,62,63].

To further validate advantages of nA-RGO film electrodes in practical application, a symmetrical solid-state supercapacitor is prepared by assembling two pieces of 25A-RGO film electrode with PVA/H_2_SO_4_ gel as the electrolyte and separator, and the ultrathin copper foil is used as current collector (Figure 7a). As shown in Figure 7b, the CV curves of the device present a roughly mirror shape at different scan rates, demonstrating an ideal capacitive behavior. The nearly symmetric GCD curves in all current densities (Figure 7c) suggest the fast response and good capacitive performance of the device [59]. The coulombic efficiencies are shown in Figure S6b. The volumetric specific capacitance of the whole device can reach 1.08 F/cm^3^ at a current density of 0.125 mA/cm^2^ (Figure 7d), which is higher than those of supercapacitors with printed graphene paper (0.13 F/cm^3^), Gold wire@RGO wires (0.41 F/cm^3^), SWCNTs/Graphene film (0.49 F/cm^3^), and Carbon onion/MnO_2_ fabric (0.68 F/cm^3^) [60,61,62,63]. As shown in Figure 7e (Ragone plot), the supercapacitor presents a volumetric energy density of 64.6 μWh/cm^3^ at a volumetric power density of 1.65 mW/cm^3^. Additionally, the CV curves at a scan rate of 20 mV/s exhibit negligible fluctuation under different deformations (bending or twisting) (Figure 7f), indicating good structural integrity and stable electrochemical performance of the device.

## 4. Conclusions

In summary, mechanically strong and flexible supercapacitor electrodes based on ANFs and RGO sheets were fabricated via vacuum-assisted filtration, followed by reduction with HI. The microstructure, electrical conductivity, mechanical and electrochemical properties of ANFs/RGO composite film electrodes are thoroughly investigated. Benefiting from hydrogen bonding and π–π interaction between ANFs and RGO sheets, the optimized ANFs/RGO film electrode shows a high tensile strength of 184.5 MPa, a Young’s modulus of 6.2 GPa, and a toughness of 5.9 MJ/m^3^. The film electrode also exhibits a volumetric specific capacitance of 134.4 F/cm^3^ (at 0.125 mA/cm^2^) and outstanding cycle life (94.6% capacitance retention after 5000 cycles). The ANFs/RGO composite film achieves an outstanding combination of mechanical and electrochemical properties. This ANFs/RGO composite film electrode will exhibit a positive prospect for the development of supercapacitors.

## Data Availability

Correspondence and requests for materials should be addressed to H. J.

## Supplementary Materials

The following are available online at https://www.mdpi.com/article/10.3390/nano13010103/s1, Figure S1: Schematic structure and digital images of the nA-RGO solid-state supercapacitor device, Figure S2: Adsorption-desorption isotherm curves of RGO, 25A-RGO and 50A-RGO, Figure S3: GCD curves at different scan rates of (a) RGO, (b) 10A-RGO and (c) 50A-RGO film electrodes, Figure S4: Areal capacitance of RGO and nA-RGO film electrodes at different current densities, Figure S5: Gravimetric capacitance of RGO and nA-RGO film electrodes at different current densities, Figure S6: Coulombic efficiency of (a) 25A-RGO film electrode and (b) supercapacitor based on 25A-RGO composite film.
