# Supplementary material for: Aramid Nanofibers/Reduced Graphene Oxide Composite Electrodes with High Mechanical Properties
Source: Nanomaterials (Basel). 2022 Dec 25;13(1):103. doi: 10.3390/nano13010103 (PMC9824775; doi:10.3390/nano13010103)
Supplement: Supplementary file 1 [file nanomaterials-13-00103-s001.zip › nanomaterials-2038156-supplementary.pdf]

Supporting Information

Aramid Nanofibers/Reduced Graphene Oxide Composite Electrodes with High Mechanical Properties

Jingyi Wang [1,2], Shaojie Lu [2], Mingyu Ye [2], Xiaowan Zhan [2], Hongbing Jia [2*], Xin Liao [2*], Antonio F. A. A. Melo [2,3,4]

[1] School of New Materials and Shoes & Clothing Engineering, Liming Vocational University, Quanzhou, 362000, China
[2] Key Laboratory for Soft Chemistry and Functional Materials of Ministry of Education, Nanjing University of Science and Technology, Nanjing 210094, China
[3] São Carlos Institute of Physics, University of São Paulo, São Carlos, São Paulo, Brazil
[4] Materials Engineering Graduate Program, Federal Institute of Education, Science and Technology of Piauí, Teresina, Brazil
* Correspondence: polymernjust@gmail.com (H.J.); liaoxin331@163.com (X.L.)

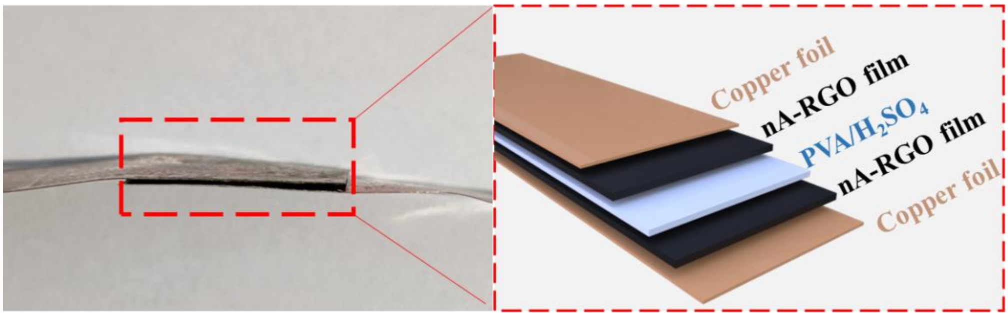

Figure S1. Schematic structure and digital images of the nA-RGO solid-state supercapacitor device

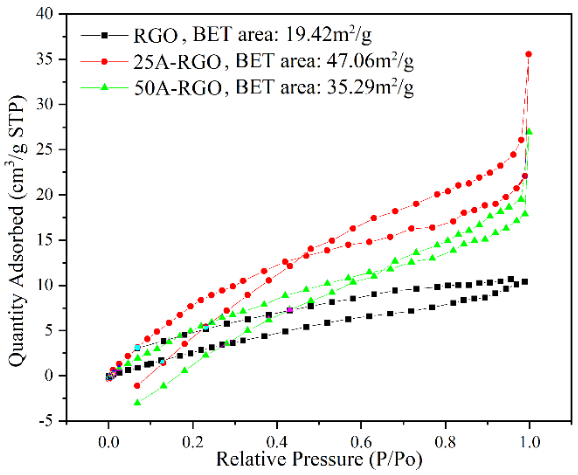

Figure S2. Adsorption-desorption isotherm curves of RGO, 25A-RGO and 50A-RGO

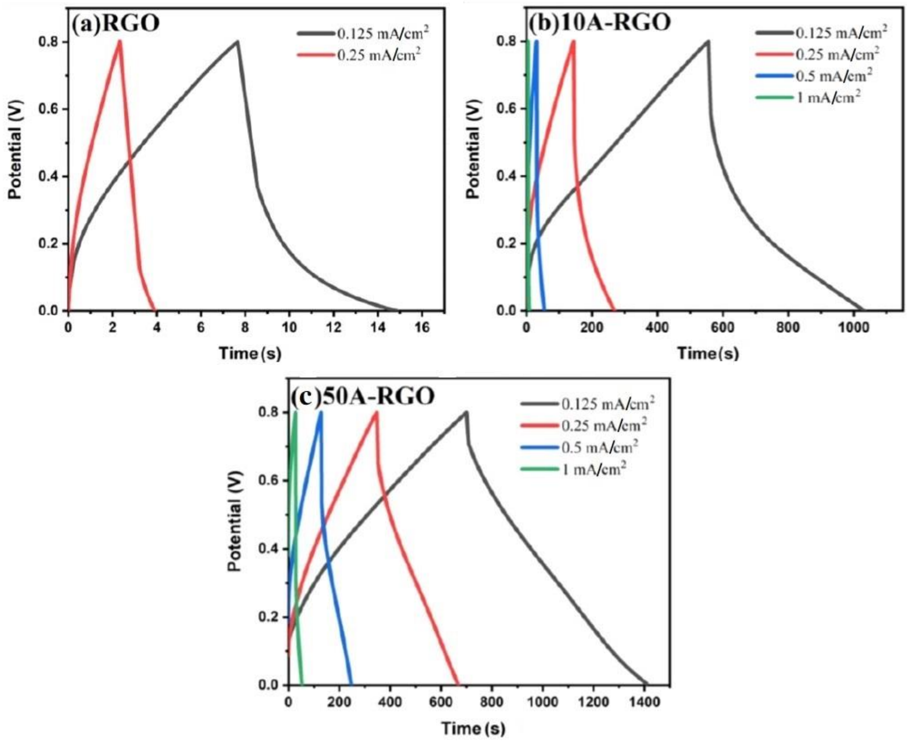

Figure S3. GCD curves at different scan rates of (**a**) RGO, (**b**) 10A-RGO and (**c**) 50A-RGO film electrodes

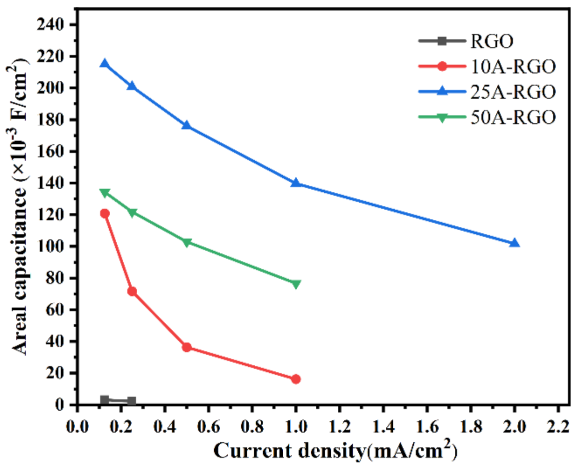

Figure S4. Areal capacitance of RGO and nA-RGO film electrodes at different current densities

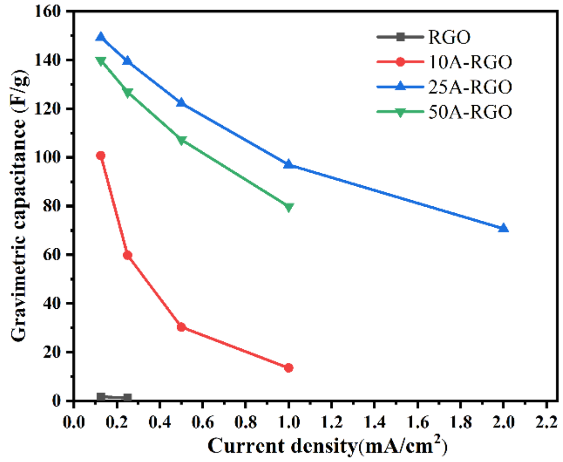

Figure S5. Gravimetric capacitance of RGO and nA-RGO film electrodes at different current densities

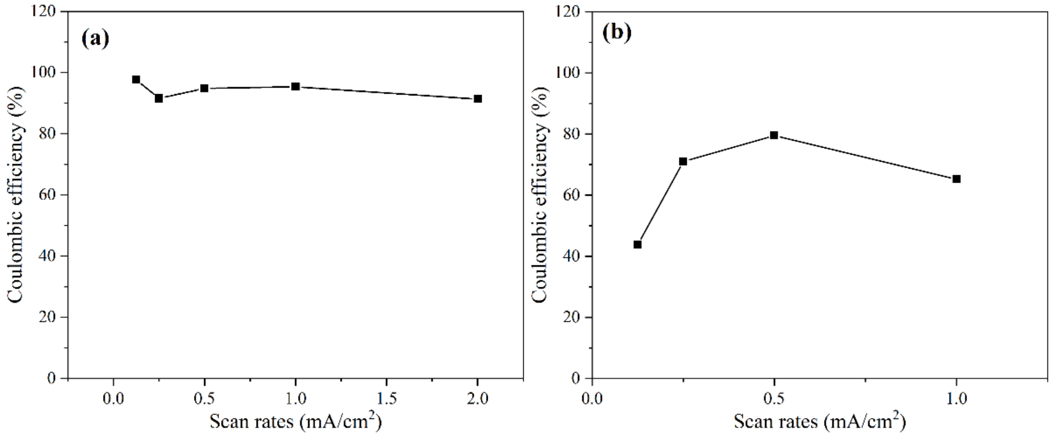

Figure S6. Coulombic efficiency of (**a**) 25A-RGO film electrode and (**b**) supercapacitor based on 25A-RGO composite film
